# Anatomic versus non-anatomic resection for early-stage intrahepatic cholangiocarcinoma: a propensity score matching and stabilized inverse probability of treatment weighting analysis

**DOI:** 10.1186/s12885-023-11341-z

**Published:** 2023-09-11

**Authors:** Qiao Ke, Lei Wang, Ziguo Lin, Hongzhi Liu, Jianying Lou, Shuguo Zheng, Xinyu Bi, Jianming Wang, Wei Guo, Fuyu Li, Jian Wang, Yamin Zheng, Jingdong Li, Shi Cheng, Weiping Zhou, Jingfeng Liu, Yongyi Zeng

**Affiliations:** 1 Department of Hepatobiliary Surgery, Mengchao Hepatobiliary Hospital of Fujian Medical University, No. 312, Xihong Road, Fuzhou, Fujian 350025 PR China; 2 Department of Hepatobiliary Surgery, Clinical Oncology School of Fujian Medical University, No. 420, Fuma Road, Fuzhou, Fujian 350014 PR China; 3 Department of Oncology, the Second Affiliated Hospital of Nanchang University, Nanchang, China; 4 Department of Hepatobiliary Surgery, the Second Hospital Affiliated to Zhejiang University, Hangzhou, China; 5 Department of Hepatobiliary Surgery, the Southwest Hospital Affiliated to the Army Medical University, Chongqing, China; 6 Department of Hepatobiliary Surgery, Cancer Hospital, Chinese Academy of Medical Sciences, Beijing, China; 7 Department of Hepatobiliary Surgery, Tongji Hospital Affiliated to Tongji Medical College, Huazhong University of Science &Technology, Wuhan, China; 8 Department of Hepatobiliary Surgery, Beijing Friendship Hospital Affiliated to Capital Medical University, Beijing, China; 9 Department of Hepatobiliary Surgery, the West China Hospital of Sichuan University, Chengdu, China; 10 Department of Hepatobiliary Surgery, Renji Hospital Affiliated to Shanghai Jiaotong University, Shanghai, China; 11 Department of Hepatobiliary Surgery, Xuanwu Hospital Affiliated to Capital Medical University, Beijing, China; 12 Department of Hepatobiliary Surgery, the Affiliated Hospital of Chuanbei Medical University, Nanchong, China; 13 Department of Hepatobiliary Surgery, Tiantan Hospital Affiliated to Capital Medical University, Beijing, China; 14 Department of Hepatobiliary Surgery III, Eastern Hepatobiliary Surgery Hospital, Naval Medical University, Shanghai, China

**Keywords:** Intrahepatic cholangiocarcinoma, Anatomic resection, Overall survival, Disease-free survival, Propensity score matching, Inverse probability of treatment weighting

## Abstract

**Background:**

Radical resection is still the most cost-effectiveness curative strategy for intrahepatic cholangiocarcinoma (ICC), but it remains controversial on the survival benefit of anatomic resection (AR). In this study, we sought to compare the oncologic outcomes between AR versus non-AR (NAR) as the primary treatment for early-stage ICC patients.

**Methods:**

Data of ICC patients who underwent hepatectomy and staged at AJCC I were retrospectively collected from 12 hepatobiliary centers in China between Dec 2012 and Dec 2015. Propensity score matching (PSM) and stabilized inverse probability of treatment weighting (IPTW) analysis were performed to minimize the effect of potential confounders, and the perioperative and long-term outcomes between AR and NAR groups were compared.

**Results:**

Two hundred seventy-eight ICC patients staged at AJCC I were eligible for this study, including 126 patients receiving AR and 152 patients receiving NAR. Compared to the NAR group, the AR group experienced more intraoperative blood loss before and after PSM or stabilized IPTW (all *P* > 0.05); AR group also experienced more intraoperative transfusion after stabilized IPTW (*P* > 0.05). In terms of disease-free survival (DFS) and overall survival (OS), no significant differences were observed between the two groups before and after PSM or stabilized IPTW (all *P* > 0.05). Multivariable Cox regression analyses found that AR was not an independent prognostic factor for either DFS or OS (all *P* > 0.05). Further analysis also showed that the survival benefit of AR was not found in any subgroup stratified by Child–Pugh grade (A or B), cirrhosis (presence or absence), tumor diameter (≤ 5 cm or > 5 cm) and pathological type (mass-forming or non-mass-forming) with all *P* > 0.05.

**Conclusion:**

Surgical approach does not influence the prognosis of patients with stage I primary ICC, and NAR might be acceptable and oncological safety.

**Supplementary Information:**

The online version contains supplementary material available at 10.1186/s12885-023-11341-z.

## Introduction

Intrahepatic cholangiocarcinoma (ICC) ranks second among the primary liver cancer, and the incidence is increasing stably globally [1, 2]. Radical resection is still the most cost-effectiveness treatment for primary ICC patients, which is preferred worldwide [1, 3]. Nonetheless, the long-term prognosis remains far from satisfactory due to the high risk of recurrence and metastasis after resection [3, 4].

Hepatectomy is the dominant part in the process of surgical resection for ICC. Generally, hepatectomy is divided into anatomic resection (AR) and non-anatomic resection (NAR) [5, 6]. Unlike NAR, where the extent of resection depends entirely on the distribution of the tumor, AR is a procedure that involves segmental resection in accordance with liver anatomy [7]. Theoretically, AR has obvious advantage over NAR in complete tumor resection and potential micrometastases eradication, which in turn results in fewer recurrence and better prognosis [8]. In a Japanese nationwide survey of 5781 hepatocellular carcinoma (HCC) patients, Eguchi et al. [9] firstly identified the advantage of AR in DFS, especially among those with tumor diameter of 2–5 cm, which was confirmed in numerous subsequent studies [10–12]. Furthermore, despite the limited and sparse literature available on this topic, reports have indicated a survival advantage of AR in patients with ICC [13, 14].

However, there are also many surgeons on the side of NAR. Radical resection without tumor residua under microscope (R0) is the main principle of hepatectomy [1, 5], and AR often involves removal of more healthy liver parenchyma, which might increase the risk of postoperative liver failure [6]. In addition, the oncologic advantage of AR was not shown with significant difference [6, 15, 16]. In an analysis 2558 solitary HCC patients, no difference was observed between AR and NAR in subgroup of tumor > 5 cm with MVI [17]. Even, the prognosis of HCC patients with cirrhosis in NAR group was better than those in AR group in a report of Japanese [18]. Likewise, Li et al. [19] have failed to find a survival benefit of AR for single ICC patients without invasion of adjacent organs or extrahepatic metastases.

One size does not fit for all. Given the evident inconsistencies in comparing AR and NAR for ICC [13, 14, 19], it is reasonable to assume the existence of a subgroup that does not benefit from AR. For instance, in cases where small HCC may not derive substantial benefits from AR, we hypothesized that the surgical approach would not significantly impact the long-term prognosis of early-stage ICC [6, 20, 21]. To investigate this further, we collected the data of ICC patients staged AJCC I from the 12 hepatobiliary centers in China and then compared the perioperative and long-term outcomes of patients receiving AR versus NAR using propensity-score matching (PSM) and stabilized inverse probability of treatment weighting (IPTW).

## Materials and methods

This study was in line with the guidance of the 1975 Declaration of Helsinki and was approved by the Institutional Review Board of Mengchao Hepatobiliary Hospital of Fujian Medical University (No. 2018_048_01). The informed consent form has been signed by all participants. Data of ICC patients who underwent hepatectomy and staged at AJCC I were retrospectively collected from 12 hepatobiliary centers in China between Dec 2012 and Dec 2015. The list of participating centers is detailed in the supplementary table 1.

### Patient selection

Patients were eligible if they met the following criteria: 1) diagnosed as ICC by pathology; 2) at least 18 years of age; 3) underwent AR or NAR treatment; 4) R0 resection; and 5) staged at I according to the 8^th^ AJCC staging system [1].

These were the exclusion criteria: 1) recurrent ICC; 2) presence of other malignancies; 3) preoperative antitumor treatment was administered; 4) death within one month after surgery, and 5) incomplete clinicopathological and/or follow-up data.

### Data collection and definitions

Clinicopathological and follow-up data were collected retrospectively, and the former including gender (female or male), age (≥ 60 or < 60 years), preoperative serum levels of CA19-9 (> 200 or ≤ 200 U/ml) and CEA (> 5 or ≤ 5 ng/ml), the Eastern Cooperative Oncology Group score (ECOG, 0–1 or ≥ 2), Child–Pugh grade (A or B), intraoperative blood loss (> 400 or ≤ 400 ml), intraoperative transfusion (yes or no), operation time (> 180 or ≤ 180 min), major hepatectomy (yes or no), surgical margin (> 1 or ≤ 1 cm), complications (yes or no), severe complications (yes or no), hospital stays (> 15 or ≤ 15 days), tumor diameter (> 5 or ≤ 5 cm), cirrhosis (yes or no), pathological type (mass-forming or non-mass-forming), tumor differentiation (well-moderate or poor), satellite (absent or present) and adjuvant therapy (yes or no). The cut-off values of the included variables were categorized by our previous reports [22].

According to the eighth edition of the TNM staging manual from the American Joint Committee on Cancer, stage I (T1N0M0) tumors do not have vascular invasion, regardless of their size, and they do not have regional lymph nodes or distant metastases [1]. R0 resection is defined as the absence of tumor residue at the surgical margin upon microscopic examination [3]. In accordance with Couinaud's classification, major hepatectomy is defined as the removal of three or more liver segments [23]. The width of SM is defined as the shortest distance between the liver section and the tumor margin [24]. The Clavien-Dindo classification was used to determine surgical complications, and severe complications were defined as Clavien-Dindo grade ≥ 3 [25]. Based on relevant guidelines or consensus [3, 23], the pathological types, liver fibrosis, tumor differentiation and presence of satellite were assessed by three pathologists independently.

### Liver resection and adjuvant therapies

AR refers to the complete removal of Couinaud’s segments determined by prior ischemia or dye staining, which is often accompanied by segmentectomy, hemihepatectomy, or trisectionectomy [26, 27]. NAR is defined as the resection of the tumor site regardless of the anatomic location, which often involved limited resection and enucleation of the tumor [26]. The regional LND was performed when metastases were suspected or diagnosed preoperatively or intraoperatively [13].

In accordance with our previous publication [28, 29], the present study encompassed various adjuvant treatment modalities, including chemotherapy, radiotherapy, and transarterial chemoembolization (TACE). Chemotherapy administration typically commenced 1–2 months following resection and consisted of 4–6 cycles. The most frequently employed chemotherapy regimens involved fluoropyrimidine-based or gemcitabine-based protocols. Adjuvant radiotherapy, on the other hand, was administered 1–2 months after resection, utilizing intensity-modulated radiation therapy with a cumulative dosage of 45–50 Gy, delivered in fractions of 1.8–2.0 Gy each. Similarly, adjuvant TACE was administered once, with a time interval ranging from three weeks to two months after the procedure. The frequently utilized TACE regimens included a combination of 5-fluorouracil (500 mg), epirubicin (20 mg), hydroxycamptothecin (10 mg), and an emulsion of lipiodol (5–10 mL). Considering the limited utilization of adjuvant treatment within our study cohort, all modalities were collectively analyzed. The decision to administer adjuvant treatment was reached through a multidisciplinary discussion, taking into account factors such as patient’s pathology, systemic condition, and individual preferences.

### Follow up and endpoints

As recommended by the ICC Chinese Expert Consensus [30], patients should be followed up every three months for two years after surgery, every six months for two to five years after surgery, and once a year after five years. Follow-up items included: 1) A general physical examination; 2) Imaging examinations: CT scan of the lungs, enhanced CT or magnetic resonance imaging (MRI) of the upper abdomen; and 3) Laboratory tests: routine blood, blood biochemistry, the serum levels of CEA and CA19-9. In the event that recurrence was confirmed, salvage treatment was initiated immediately.

The primary endpoints were the DFS and OS. DFS was defined as the time from resection to recurrence, while OS was defined as the time from resection to death or last follow-up.

### Statistical analysis

Since variables included in this study were all categorical, they all be expressed as number and percentages and differences between the two groups were assessed by the Chi-square test or Fisher's exact test. The Kaplan–Meier method was used to analyze DFS and OS, while the log-rank test was used for between-group comparisons. A Cox proportional hazards model was used to determine risk factors for DFS and OS, and variables with *P* value less than 0.1 were included in the multivariate analysis. Of note, in multivariate analysis, liver resection types were automatically included regardless of their differences in univariate analysis.

To overcome selection bias resulting from unbalanced baseline characteristics between groups, we performed matching analyses based on preoperative variables using the PSM and stabilized IPTW methods. PSM was used with a 1:1 nearest neighbor method and a caliper value of 0.01. The stabilized IPTW was generated with propensity score (PS) within a pseudo-data set. The PS was estimated using a multivariable logistic regression model with liver resection types as the dependent variable and other clinically relevant confounders as covariates (including gender, age, CA19-9, CEA, ECOG score, Child–Pugh class, major hepatectomy, cirrhosis, surgical margin, tumor diameter, pathological type, tumor differentiation, satellite, adjuvant therapy). Stabilized weight (SW) formula was AR group weight = pt/PS and NAR group weight = 1-pt/(1-PS), where pt represents the probability of AR group without accounting for covariates and PS represents propensity score.

Statistical analyses were conducted using R software (version 4.1.1, R Foundation), and statistical significance was determined by a two side *P* value less than 0.05.

## Results

This study's flow chart was shown in supplementary Fig. 1. Between December 2012 and December 2015, data on 501 consecutive patients diagnosed with ICC and undergoing hepatectomy were collected. A total of 278 patients with AJCC stage I were eligible for this study, of whom 126 (45.3%) received AR (AR group) and 152 (54.7%) received NAR (NAR group). The PSM generated 58 matched pairs of patients in the AR and NAR groups, while the stabilized IPTW generated 127 patients in the AR group and 145 patients in the NAR group.

### Baseline characteristics

The baseline characteristics were summarized in Table 1. Before PSM, in comparison to the NAR group, AR group had a greater percentage of female patients, age ≥ 60 years, CEA > 5 ng/ml, Child–Pugh class A, surgical margin ≥ 1 cm, hospital stay > 15 days, tumor diameter > 5 cm, pathological type of mass-forming, but a lower percentage of ECOG score ≥ 2 and cirrhosis (all *P* < 0.05). Among other variables, the proportion of CA19-9 > 200U/ml, major hepatectomy, tumor differentiation, satellites and adjuvant therapy were comparable between the two groups (all *P* > 0.05). As was expected, no significant difference was found between the two groups in baseline characteristics after PSM and stabilized IPTW (all *P* > 0.05).

**Table 1:** Basic clinicopathological characteristics between the AR and NAR groups before and after PSM or stabilized IPTW

Variables		Entire cohort			PSM cohort			Stabilized IPTW cohort		
		**AR**	**NAR**	***P*-Value**	**AR**	**NAR**	***P*-Value**	**AR**	**NAR**	***P*-Value**
		(*n* = 126)	(*n* = 152)		(*n* = 58)	(*n* = 58)		(*n* = 127)	(*n* = 145)	
**Gender**	Female	58 (46.0%)	41 (27.0%)	0.001	20 (34.5%)	19 (32.8%)	1	44 (34.6%)	48 (33.1%)	0.789
	Male	68 (54.0%)	111 (73.0%)		38 (65.5%)	39 (67.2%)		83 (65.4%)	97 (66.9%)	
**Age**	< 60 years	65 (51.6%)	99 (65.1%)	0.031	36 (62.1%)	35 (60.3%)	1	78 (61.4%)	89 (61.4%)	0.995
	≥ 60 years	61 (48.4%)	53 (34.9%)		22 (37.9%)	23 (39.7%)		49 (38.6%)	56 (38.6%)	
**CA19-9**	≤ 200 U/mL	103 (81.7%)	137 (90.1%)	0.064	50 (86.2%)	46 (79.3%)	0.461	110 (86.6%)	126 (86.9%)	0.945
	> 200 U/mL	23 (18.3%)	15 (9.9%)		8 (13.8%)	12 (20.7%)		17 (13.4%)	19 (13.1%)	
**CEA**	≤ 5 ng/mL	89 (70.6%)	133 (87.5%)	< 0.001	44 (75.9%)	44 (75.9%)	1	102 (80.3%)	119 (82.0%)	0.712
	> 5 ng/mL	37 (29.4%)	19 (12.5%)		14 (24.1%)	14 (24.1%)		25 (19.7%)	26 (17.9%)	
**ECOG score**	0–1	61 (48.4%)	48 (31.6%)	0.006	27 (46.6%)	23 (39.7%)	0.574	52 (40.9%)	56 (38.6%)	0.696
	≥ 2	65 (51.6%)	104 (68.4%)		31 (53.4%)	35 (60.3%)		75 (59.1%)	89 (61.4%)	
**Child–Pugh class**	A	86 (68.3%)	80 (52.6%)	0.012	34 (58.6%)	33 (56.9%)	1	76 (59.8%)	84 (57.9%)	0.749
	B	40 (31.7%)	72 (47.4%)		24 (41.4%)	25 (43.1%)		51 (40.2%)	61 (42.1%)	
**Major hepatectomy**	No	51 (40.5%)	72 (47.4%)	0.303	25 (43.1%)	27 (46.6%)	0.852	62 (48.8%)	66 (45.5%)	0.586
	Yes	75 (59.5%)	80 (52.6%)		33 (56.9%)	31 (53.4%)		65 (51.2%)	79 (54.5%)	
**Cirrhosis**	No	95 (75.4%)	84 (55.3%)	< 0.001	36 (62.1%)	37 (63.8%)	1	81 (63.8%)	91 (62.8%)	0.862
	Yes	31 (24.6%)	68 (44.7%)		22 (37.9%)	21 (36.2%)		46 (36.2%)	54 (37.2%)	
**Surgical margin**	< 1 cm	58 (46.0%)	96 (63.2%)	0.006	29 (50.0%)	29 (50.0%)	1	68 (53.5%)	80 (55.2%)	0.788
	≥ 1 cm	68 (54.0%)	56 (36.8%)		29 (50.0%)	29 (50.0%)		59 (46.5%)	65 (44.8%)	
**Tumor diameter**	≤ 5 cm	44 (34.9%)	72 (47.4%)	0.049	24 (41.4%)	27 (46.6%)	0.708	59 (46.5%)	66 (45.5%)	0.877
	> 5 cm	82 (65.1%)	80 (52.6%)		34 (58.6%)	31 (53.4%)		68 (53.5%)	79 (54.5%)	
**Mass-forming**	No	30 (23.8%)	56 (36.8%)	0.027	16 (27.6%)	20 (34.5%)	0.547	41 (32.3%)	48 (33.1%)	0.886
	Yes	96 (76.2%)	96 (63.2%)		42 (72.4%)	38 (65.5%)		86 (67.7%)	97 (66.9%)	
**Tumor differentiation**	Well&Moderate	94 (74.6%)	121 (79.6%)	0.397	42 (72.4%)	40 (69.0%)	0.838	98 (77.2%)	114 (78.6%)	0.772
	Poor	32 (25.4%)	31 (20.4%)		16 (27.6%)	18 (31.0%)		29 (22.8%)	31 (21.4%)	
**Satellite**	No	113 (89.7%)	146 (96.1%)	0.063	57 (98.3%)	56 (96.6%)	1	118 (92.9%)	138 (95.2%)	0.594
	Yes	13 (10.3%)	6 (3.9%)		1 (1.7%)	2 (3.4%)		9 (7.1%)	7 (4.8%)	
**Adjuvant Therapy**	No	100 (79.4%)	128 (84.2%)	0.373	52 (89.7%)	49 (84.5%)	0.406	101 (79.5%)	116 (80.0%)	0.956
	Yes	26 (20.6%)	24 (15.8%)		6 (10.3%)	9 (15.5%)		26 (20.5%)	29 (20.0%)	

*Notes*: *PSM* Propensity score matching, *IPTW* Inverse probability of treatment weighting, *NAR* Nonanatomic resection, *AR* Anatomic resection, *HBV* Hepatitis B virus, *CA19-9* Carbohydrate antigen 19–9, *CEA* Carcinoembryonic antigen, *ECOG* The Eastern Cooperative Oncology Group

### Perioperative outcomes

As shown in Table 2, the proportion of intraoperative blood loss > 400 mL, intraoperative transfusions, operation time > 180 min and hospital stays > 15 days were significantly higher in the AR group compared to the NAR group (all *P* < 0.05); however, there were no significant differences observed in the incidence of complications or severe complications in the crude cohorts (both *P* > 0.05). Additionally, the AR group had higher rates of intraoperative blood loss > 400 mL in both the PSM and stabilized IPTW cohorts (both *P* < 0.05), as well as a higher rate of intraoperative transfusion in the stabilized IPTW cohort (*P* < 0.05). Nonetheless, neither group experienced postoperative liver failure, and the incidence of complications and severe complications, as well as hospital stays, was comparable between the two groups (all *P* > 0.05).

**Table 2:** Perioperative and postoperative outcomes between the AR and NAR groups before and after PSM or stabilized IPTW

Variables		Entire cohort			PSM cohort			Stabilized IPTW cohort		
		**AR**	**NAR**	***P*-Value**	**AR**	**NAR**	***P*-Value**	**AR**	**NAR**	***P*-Value**
		(*n* = 126)	(*n* = 152)		(*n* = 58)	(*n* = 58)		(*n* = 127)	(*n* = 145)	
**Intraoperative blood loss**	≤ 400 mL	89 (70.6%)	136 (89.5%)	< 0.001	43 (74.1%)	52 (89.7%)	0.030	92 (72.4%)	129 (89.0%)	< 0.001
	> 400 mL	37 (29.4%)	16 (10.5%)		15 (25.9%)	6 (10.3%)		35 (27.6%)	16 (11.0%)	
**Intraoperative transfusion**	No	93 (73.8%)	140 (92.1%)	< 0.001	45 (77.6%)	52 (89.7%)	0.079	99 (78.0%)	130 (89.7%)	0.008
	Yes	33 (26.2%)	12 (7.9%)		13 (22.4%)	6 (10.3%)		28 (22.0%)	15 (10.3%)	
**Operation time**	≤ 180 min	61 (48.4%)	94 (61.8%)	0.025	30 (51.7%)	34 (58.6%)	0.455	66 (52.0%)	80 (55.2%)	0.597
	> 180 min	65 (51.6%)	58 (38.2%)		28 (48.3%)	24 (41.4%)		61 (48.0%)	65 (44.8%)	
**Hospital stays**	≤ 15 days	59 (46.8%)	105 (69.1%)	< 0.001	28 (48.3%)	34 (58.6%)	0.264	69 (54.3%)	95 (65.5%)	0.060
	> 15 days	67 (53.2%)	47 (30.9%)		30 (51.7%)	24 (41.4%)		58 (45.7%)	50 (34.5%)	
**Complications**	No	95 (75.4%)	122 (80.3%)	0.406	44 (75.9%)	46 (79.3%)	0.656	98 (77.2%)	114 (78.6%)	0.773
	Yes	31 (24.6%)	30 (19.7%)		14 (24.1%)	12 (20.6%)		29 (22.8%)	31 (21.4%)	
**Severe Complications**	No	112 (88.9%)	137 (90.1%)	0.736	52 (89.7%)	53 (91.4%)	0.751	113 (89.0%)	130 (89.7%)	0.856
	Yes	14 (11.1%)	15 (9.9%)		6 (10.3%)	5 (8.6%)		14 (11.0%)	15 (10.3%)	

*Notes*: *PSM* Propensity score matching, *IPTW* Inverse probability of treatment weighting, *NAR* Nonanatomic resection, *AR* Anatomic resection

### Long-term outcomes

**Fig. 1:**
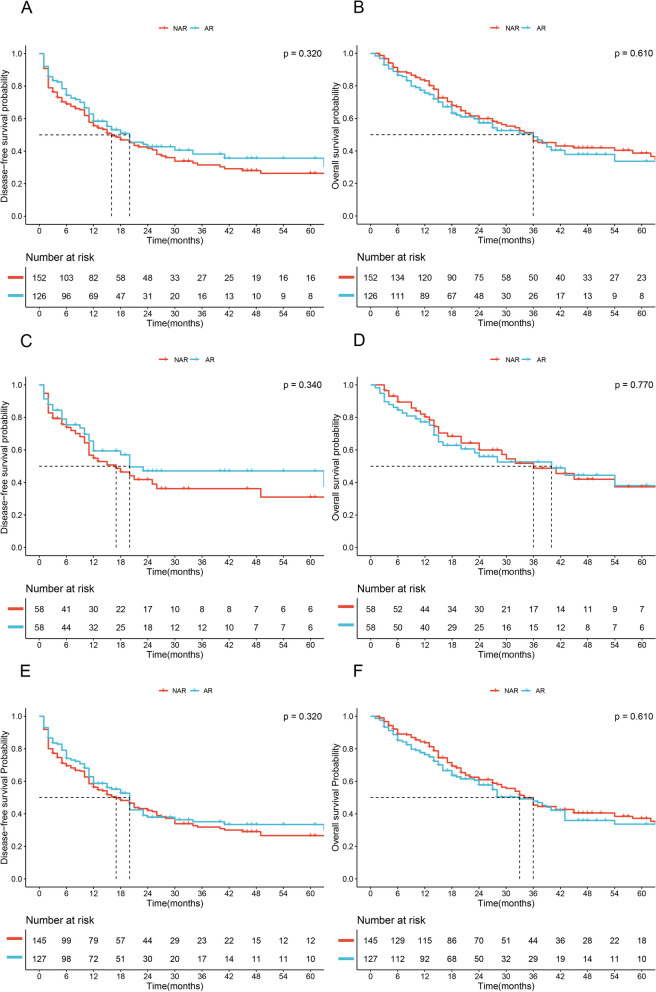
Comparison of disease-free survival and overall survival between AR and NAR groups (in entire cohort: **A**, disease-free survival; **B**, overall survival; in PSM cohort: **C**, disease-free survival; **D**, overall survival; in stabilized IPTW cohort: **A**, disease-free survival; **B**, overall survival)

In the crude cohort, the median DFS was longer in the AR group than in the NAR group (20 months vs. 16 months, respectively); however, this difference was not statistically significant (*P* = 0.320, Fig. 1A). Additionally, the median OS was comparable among both groups (36 months vs. 36 months, *P* = 0.610, Fig. 1B). In the PSM cohort, the AR group showed a slight advantage in median DFS and OS over the NAR group (DFS: 20 months vs. 17 months; OS: 40 months vs. 36 months; respectively), but neither of the differences were statistically significant (*P* = 0.340, *P* = 0.770; Fig. 1C and D, respectively). The stabilized IPTW cohort also demonstrated similar results between the two groups (DFS: 20 months vs. 17 months, *P* = 0.320; OS: 33 months vs. 36 months, *P* = 0.610; Fig. 1E and F, respectively).

The postoperative recurrence patterns between the two groups were compared within the entire cohort, and the results are presented in Table 3. Over the course of the follow-up period, it was observed that 53.2% (67/126) of patients in the AR group experienced recurrence, while 63.8% (97/152) of patients in the NAR group experienced recurrence. Patients in the NAR group demonstrated a tendency towards higher recurrence rates in various locations, including intrahepatic sites (such as the resection margin, adjacent segment, and distant segment) and extrahepatic sites (including single metastasis and multiple metastases), as well as both intra- and extrahepatic sites. However, these differences did not reach statistical significance (all *P* > 0.05).

**Table 3:** Postoperative recurrence patterns of AR and NAR groups in entire cohort

**Recurrence Patterns**		**AR**	**NAR**	***P*-Value**
		(*n* = 126)	(*n* = 152)	
Intrahepatic	Resection margin	5 (4.0%)	9 (5.9%)	0.782
	Adjacent segment	26 (20.6%)	38 (25.0%)	
	Distant segment	20 (15.9%)	24 (15.8%)	
Extrahepatic	Single metastasis	5 (4.0%)	7 (4.6%)	1
	Multiple metastases	4 (3.2%)	8 (5.3%)	
Intra- and extrahepatic		7 (5.6%)	11 (7.2%)	0.747

*Notes*: *NAR* Nonanatomic resection, *AR* Anatomic resection

### Prognostic factors analysis

Data on univariate and multivariate Cox regression analysis for DFS and OS in the three cohorts are shown in Tables 4 and 5 and Supplementary Table 2. The results indicate that surgical approach (AR vs. NAR) was not an independent prognostic factor for DFS or OS (all P > 0.05).

**Table 4:** Univariate and multivariate analysis of disease-free survival and overall survival in PSM cohort

Characteristics	Disease-free survival				Overall survival			
	**Univariate**		**Multivariate**		**Univariate**		**Multivariate**	
	**HR (95CI)**	***P-*value**	**HR (95CI)**	***P-*value**	**HR (95CI)**	***P-*value**	**HR (95CI)**	***P-*value**
**Gender (**Male vs Female**)**	0.68 (0.39–1.18)	0.173			0.67 (0.37–1.21)	0.184		
**Age (**≥ 60 vs < 60 years**)**	1.25 (0.76–2.08)	0.380			1.23 (0.72–2.11)	0.444		
**CA19-9 (**> 200 vs ≤ 200 U/mL)	1.03 (0.52–2.03)	0.927			1.41 (0.74–2.68)	0.293		
**CEA (**> 5 vs ≤ 5 ug/mL)	0.90 (0.49–1.67)	0.743			1.77 (1.01–3.12)	0.047	1.84 (1.02–3.38)	0.044
**ECOG score (**≥ 2 vs 0–1**)**	1.04 (0.63–1.72)	0.886			1.15 (0.66–1.98)	0.623		
**Child–Pugh class** (B vs A**)**	1.40 (0.85–2.31)	0.185			1.69 (0.99–2.88)	0.053	1.51 (0.87–2.61)	0.144
**Intraoperative blood loss** (> 400 vs ≤ 400 mL**)**	1.01 (0.68–1.49)	0.968			0.92 (0.46–1.84)	0.824		
**Intraoperative transfusion (**Yes vs No**)**	0.83 (0.43–1.59)	0.568			1.18 (0.58–2.42)	0.645		
**Operation time (**> 180 vs ≤ 180 min**)**	1.13 (0.72–1.48)	0.374			1.24 (0.88–1.76)	0.783		
**Major hepatectomy (**Yes vs No**)**	1.45 (0.87–2.41)	0.155			2.16 (1.22–3.84)	0.008	2.04 (1.18–3.64)	0.011
**Surgical margin (**≥ 1 cm vs < 1 cm**)**	0.55 (0.33–0.91)	0.020	0.63 (0.37–0.97)	0.033	0.62 (0.36–1.06)	0.083	0.62 (0.35–1.10)	0.099
**Anatomic resection (**Yes vs No**)**	0.79 (0.48–1.30)	0.348	0.81 (0.49–1.34)	0.415	1.08 (0.64–1.83)	0.775	0.97 (0.56–1.69)	0.920
**Hospital stays (**> 15 vs ≤ 15 days**)**	1.04 (0.63–1.74)	0.870			1.21 (0.71–2.06)	0.484		
**Cirrhosis (**Yes vs No**)**	0.80 (0.47–1.35)	0.400			0.82 (0.47–1.43)	0.485		
**Tumor diameter (**> 5 vs ≤ 5 cm**)**	1.37(0.83–2.28)	0.223			2.08 (1.17–3.69)	0.012	1.98 (1.11–3.55)	0.021
**Mass-forming (**Yes vs No**)**	0.63 (0.38–1.06)	0.080	0.75 (0.44–1.26)	0.273	0.78 (0.45–1.34)	0.370		
**Tumor differentiation (**Well&Moderate vs Poor**)**	0.57 (0.32–1.05)	0.070	0.68 (0.36–1.28)	0.231	0.94 (0.52–1.68)	0.827		
**Satellite (**Yes vs No**)**	0.67 (0.09–4.86)	0.694			2.37 (0.57–9.83)	0.234		
**Adjuvant Therapy (**Yes vs No**)**	1.20 (0.61–2.37)	0.599			0.83 (0.37–1.84)	0.644		

*Note*: *PSM* Propensity score matching, *NAR* Nonanatomic resection, *AR* Anatomic resection, *HBV* Hepatitis B virus, *CA19-9* Carbohydrate antigen 19–9, *CEA* Carcinoembryonic antigen, *ECOG* The Eastern Cooperative Oncology Group

**Table 5:** Univariate and multivariate analysis of disease-free survival and overall survival in stabilized IPTW cohort

Characteristics	Disease-free survival				Overall survival			
	**Univariate**		**Multivariate**		**Univariate**		**Multivariate**	
	**HR (95CI)**	***P-*value**	**HR (95CI)**	***P-*value**	**HR (95CI)**	***P-*value**	**HR (95CI)**	***P-*value**
**Gender (**Male vs Female**)**	0.73 (0.50–1.10)	0.098	0.81 (0.57–1.20)	0.260	0.75 (0.50–1.10)	0.150		
**Age (**≥ 60 vs < 60 years**)**	0.89 (0.64–1.30)	0.510			1.10 (0.73–1.50)	0.790		
**CA19-9 (**> 200 vs ≤ 200 U/mL**)**	0.91 (0.56–1.50)	0.700			1.10 (0.70–1.70)	0.700		
**CEA (**> 5 vs ≤ 5 ug/mL**)**	0.80 (0.50–1.30)	0.340			1.20 (0.80–1.90)	0.370		
**ECOG score (**≥ 2 vs 0–1**)**	0.74 (0.53–1.11)	0.190			0.93 (0.64–1.30)	0.680		
**Child–Pugh class (**B vs A**)**	1.20 (0.89–1.70)	0.210			1.20 (0.86–1.80)	0.250		
**Intraoperative blood loss (**> 400 vs ≤ 400 mL**)**	0.84 (0.55–1.30)	0.430			0.77 (0.45–1.30)	0.350		
**Intraoperative transfusion (**Yes vs No)	0.93 (0.58–1.50)	0.750			0.97 (0.57–1.70)	0.910		
**Operation time (**> 180 vs ≤ 180 min**)**	0.86 (0.51–1.23)	0.552			1.12 (0.88–1.30)	0.443		
**Major hepatectomy (**Yes vs No**)**	1.30 (0.92–1.80)	0.130			1.70 (1.20–2.50)	0.005	1.70 (0.59–4.80)	0.340
**Surgical margin (**≥ 1 cm vs < 1 cm**)**	0.53 (0.37–0.75)	< 0.001	0.52 (0.36–0.77)	0.001	0.63 (0.43–0.91)	0.015	0.93 (0.72–1.20)	0.600
**Anatomic resection (**Yes vs No**)**	0.90 (0.64–1.30)	0.550	0.87 (0.62–1.20)	0.430	1.10 (0.75–1.60)	0.620	1.30 (0.96–1.60)	0.091
**Hospital stays (**> 15 vs ≤ 15 days**)**	1.30 (0.91–1.80)	0.160			1.20 (0.82–1.70)	0.350		
**Cirrhosis (**Yes vs No**)**	0.97 (0.69–1.40)	0.880			1.10 (0.73–1.60)	0.740		
**Tumor diameter (**> 5 vs ≤ 5 cm**)**	1.30 (0.89–1.70)	0.190			1.70 (1.20–2.50)	0.006	1.80 (1.28–2.30)	0.009
**Mass-forming (**Yes vs No**)**	0.64 (0.45–0.89)	0.009	0.65 (0.46–0.92)	0.014	0.74 (0.51–1.10)	0.110		
**Tumor differentiation (**Well&Moderate vs Poor**)**	0.73 (0.48–1.10)	0.160			1.00 (0.64–1.60)	0.990		
**Satellite (**Yes vs No**)**	1.80 (1.01–3.20)	0.047	2.00 (1.20–3.50)	0.013	2.10 (1.10–4.20)	0.031	1.50 (0.81–2.90)	0.190
**Adjuvant Therapy (**Yes vs No**)**	0.97 (0.67–1.40)	0.870			0.87 (0.55–1.40)	0.540		

*Note*: *IPTW* Inverse probability of treatment weighting, *NAR* Nonanatomic resection, *AR* Anatomic resection, *HBV* Hepatitis B virus, *CA19-9* Carbohydrate antigen 19–9, *CEA* Carcinoembryonic antigen, *ECOG* The Eastern Cooperative Oncology Group

In the crude cohort, multivariate analysis revealed that surgical margin ≥ 1 cm (HR = 0.63, 95%CI = 0.46–0.88, *P* = 0.007) and pathological type of mass-forming (HR = 0.71, 95%CI = 0.51–0.97, *P* = 0.034) were independent prognostic factors for DFS, and surgical margin ≥ 1 cm (HR = 0.68, 95%CI = 0.47–0.99, *P* = 0.042) and satellite (HR = 2.36, 95%CI = 1.24–4.51, *P* = 0.009) were independent prognostic factors for OS (Supplementary Table 2).

In the PSM cohort, surgical margin ≥ 1 cm (HR = 0.63, 95%CI = 0.37–0.97, *P* = 0.033) was found to be an independent prognostic factor for DFS (Table 3), while CEA > 5ug/mL (HR = 1.84, 95%CI = 1.02–3.38, *P* = 0.044), major hepatectomy (HR = 2.04, 95%CI = 1.18–3.64, *P* = 0.011) and tumor diameter > 5 cm (HR = 1.98, 95%CI = 1.11–3.55, *P* = 0.021) were identified as independent prognostic factors for OS (Table 3).

In the stabilized IPTW cohort, surgical margin ≥ 1 cm (HR = 0.52, 95%CI = 0.36–0.77, *P* = 0.001), pathological type of mass-forming (HR = 0.65, 95%CI = 0.46–0.92, *P* = 0.014) and satellite (HR = 2.00, 95%CI = 1.20–3.50, *P* = 0.013) were found to be independent prognostic factors for DFS (Table 4), while tumor diameter > 5 cm (HR = 1.80, 95%CI = 1.28–2.30, *P* = 0.009) was identified as independent prognostic factor for OS (Table 4).

### Subgroup analysis stratified by different risk factors

**Fig. 2:**
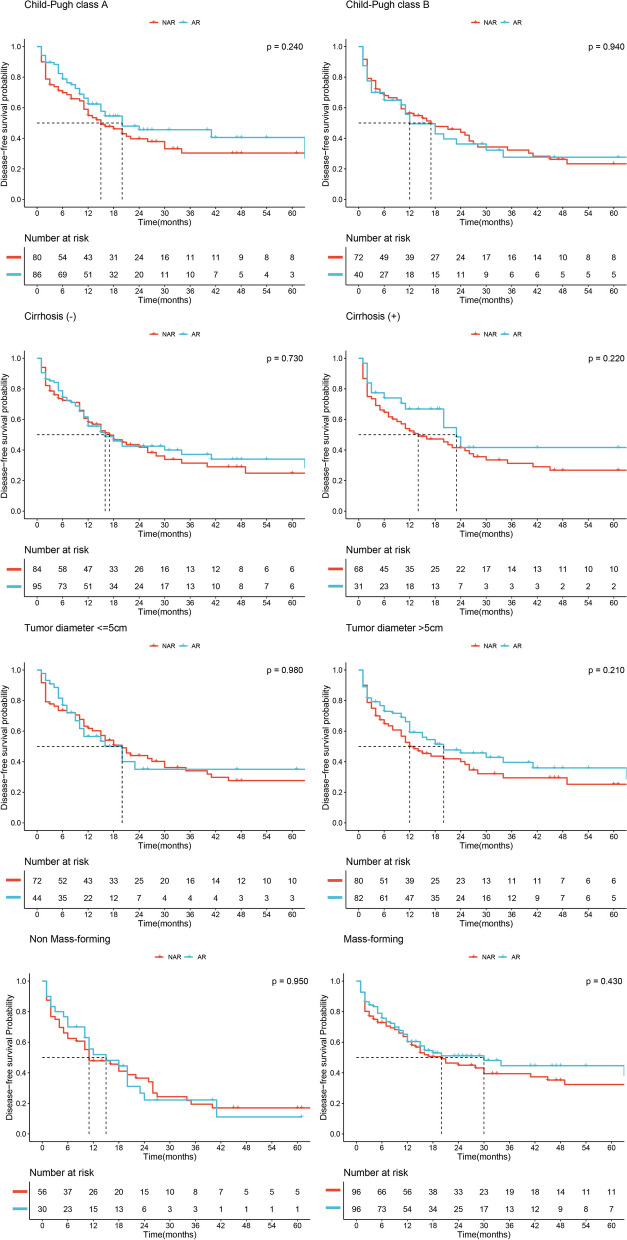
Disease-free survival of AR and NAR groups stratified by different potential confounders in entire cohort

**Fig. 3:**
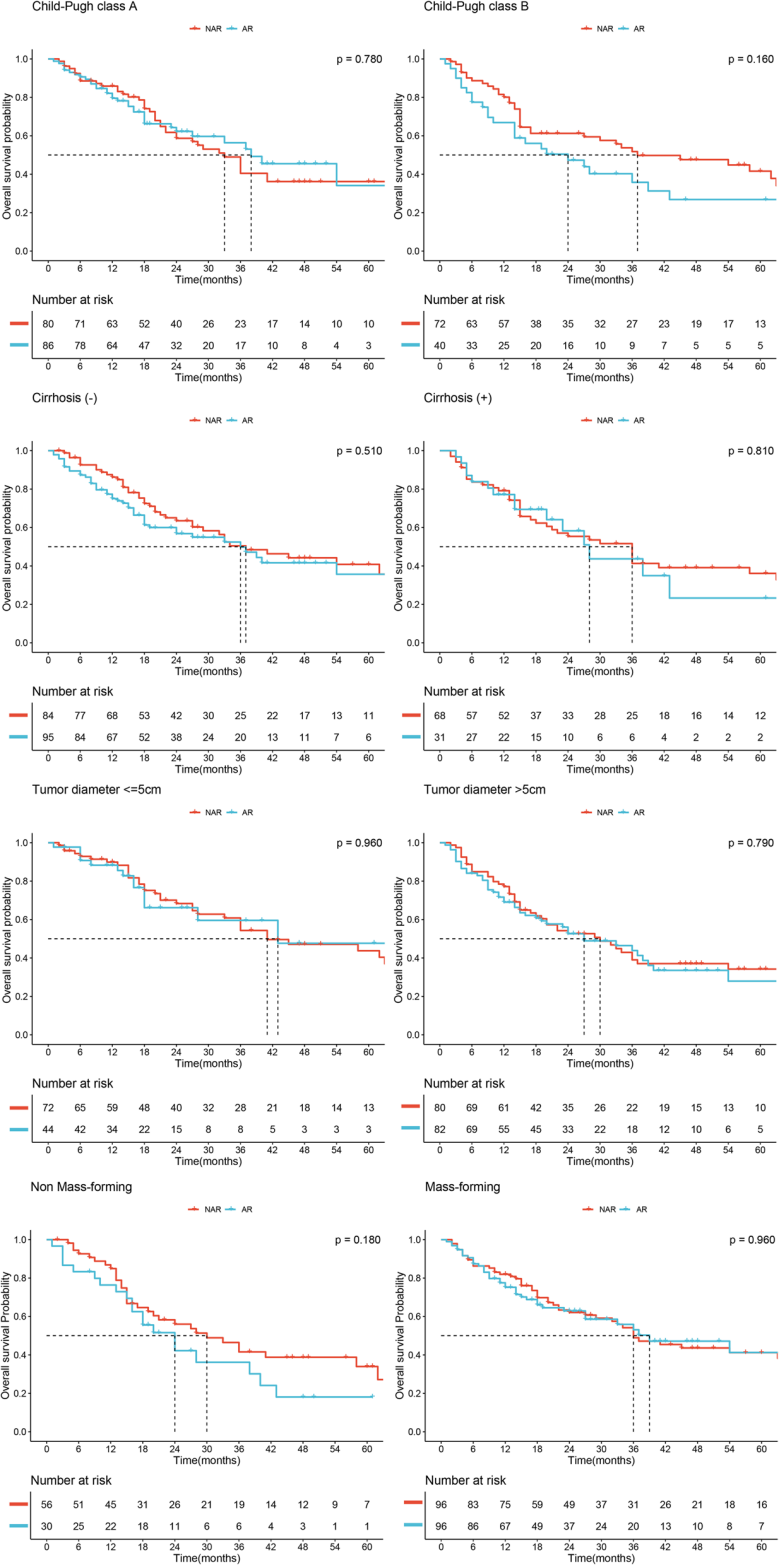
Overall survival of AR and NAR groups stratified by different potential confounders in entire cohort

In the entire cohort, we conducted subgroup analyses based on Child–Pugh class (A vs. B), presence or absence of cirrhosis, tumor diameter (> 5 cm vs. ≤ 5 cm) and pathological type (mass-forming vs. non-mass-forming). Kaplan–Meier survival curves were used to evaluate DFS within these subgroups, and no significant advantage of the AR group over the NAR group was observed (all *P* > 0.05, Fig. 2). Furthermore, in terms of OS within these subgroups, the AR group did not demonstrate any superiority over the NAR group (all *P* > 0.05, Fig. 3).

### Literature review

A comprehensive literature search was conducted in databases including PubMed, Embase, the Cochrane Library, Medline, and Web of Science to identify relevant studies evaluating the potential benefits of AR for ICC patients. Ultimately, as presented in Supplementary Table 3, a total of six studies encompassing 1523 patients were included in the analysis [13, 14, 19, 31–33], with 764 patients allocated to the AR group and 759 patients allocated to the NAR group. The findings from five studies collectively indicated a favorable effect of AR on the prognosis of ICC, along with reduced rates of postoperative recurrence [13, 14, 31–33]. Conversely, the outcomes of the remaining study indicated that the implementation of AR did not yield improvements in prognosis among patients with solitary ICC that lacked direct invasion of adjacent organs or extrahepatic metastases [19]. In fact, this specific subgroup of patients experienced worse outcomes with AR compared to NAR [19]. Notably, it should be mentioned that a majority of the aforementioned studies were conducted at single centers, and there were substantial differences in baseline characteristics observed across some studies.

## Discussion

In this study, we comprehensively evaluated the oncologic outcomes of AR in ICC patients with AJCC stage I from 12 institutions from China. In accordance with our hypothesis, we observed no statistically significant differences in DFS and OS between the AR and NAR groups. Furthermore, these results remained largely consistent even after PSM and stabilized IPTW. Additionally, none of the subgroups stratified by variables such as Child–Pugh grade, cirrhosis, tumor size, and pathological type demonstrated a survival advantage associated with AR.

Hepatectomy has lived through from the early wedge resection, lobectomy, irregular partial hepatectomy, to the present AR technique [34]. As early as 1985, Makuuchi et al. [27] introduced the concept of AR and it has become widely used in clinical practice in recent years. Compared with NAR, AR is more extensive and allows the removal of the tumor as well as any potential intrahepatic metastases, thereby reducing postoperative recurrence rates and improving long-term survival [35, 36]. In addition, AR has the potential to reduce intraoperative bleeding and postoperative complications despite its controversy [8, 36]. However, in clinical practice, AR is still limited by other factors such as liver function, residual liver volume, tumor location, tumor number and diameter.

It is the major concern of HPB surgeons whether AR could bring oncological benefit. In a recent systematic review, AR was confirmed to be superior to NAR in DFS and OS of HCC patients [8]; however, the conclusion was weakened by the heterogeneity within studies, such as liver function and tumor stage. In the latest matched study, AR was found to be no better than NAR for HCC patients within Milan criteria [15]. In a retrospective study of 702 patients, Si et al. [13] performed subgroup analyses according to different AJCC stages and concluded that AR was beneficial only to ICC patients with stage IB or II tumors without vascular invasion; however, the study was conducted only at one institution, which limited its generalizability. In contrast, Li et al. [19] found that AR did not improve the prognosis in solitary ICC in a PSM analysis, but the sample size was small and subgroup analyses were not performed. In this study, we enrolled 12 more centers distributed in China to decrease the regional bias, and then only included patients staged at AJCC stage I to decrease the effect of potential confounders. In addition, PSM and stablized IPSW were also adopted to overcome selection bias resulting from unbalanced baseline characteristics between groups. The survival advantage of AR over NAR was observed in none of the three cohorts; therefore, we concluded with credibility that AR could not bring survival benefit to early-stage ICC patients.

Further, we also conducted subgroup analysis to decrease the effect of potential confounding factors on the results in the present study. Liver function is the first concern in the decision-making of hepatectomy, and sufficient FLR is the prerequisite of AR [37]. In this study, subgroup analysis stratified by liver function showed no survival difference between AR and NAR either in subgroup of Child–Pugh grade A or in subgroup of Child–Pugh grade B. Second, the presence of cirrhosis is also an influential factor in determining whether AR could be performed, and patients with this condition may be at an increased risk of postoperative liver failure [38]. Likewise, the survival advantage of AR was not shown in subgroups stratified by cirrhosis in the present study. Third, tumor diameter is the most intuitive metric in the clinical treatment decision, and larger tumor across the lobes is also a relative contraindication of AR [37]. Similarly, no difference was observed between AR and NAR in DFS and OS among patients with tumor diameter ≤ 5 cm or > 5 cm. Fourth, the type of pathology represents a significant prognostic factor [30], and thus we conducted a subgroup analysis. However, the results did not indicate any discernible benefit of AR for either mass-forming or non-mass-forming types. All these findings above indicated that the conclusion that AR had no survival advantage in ICC patients with stage I was robust, and would not be affected by the other clinical factors.

Finally, safety is the bottom line of surgeons. Postoperative liver failure followed by AR has always been circling in the head of HPB surgeons regardless of progress in real-time navigation and indocyanine green fluorescence imaging [39]. In this study, we found that neither AR group nor NAR group experienced postoperative liver failure. With the advancement of perioperative management and surgical instrumentation [39, 40], standardized and procedural AR are becoming increasingly popular in clinical practice. In this study, AR did not increase the risk of complication and prolong hospital stay. However, the fact that AR increased the risk of intraoperative blood loss > 400 ml was observed in all the three cohorts (all *P* < 0.05), indicating caution should be exercised when making the decision to perform AR.

This study had some drawbacks, although it provided convincing evidences for decision-making for hepatectomy. First, selection bias and recall bias are inherent defects of retrospective studies, although PSM and stabilized IPTW were performed. Second, we selected a very small span of study time from 2010 to 2015 to decrease the potential treatment bias, but surgery bypass and extension are not available, all of which might have effects on prognosis. Third, other clinical factors such as MVI are also the key decision-making factors, but the incidence of MVI was too low to conduct corresponding subgroup analysis. Considering the low incidence of ICC, multicenter, randomized, and prospective studies, further meta-analyses are warranted.

## Conclusion

We concluded that early-stage ICC patients would not be benefited from AR, and the survival advantage of AR was not shown in none of the subgroups stratified by Child–Pugh grade, surgical margin, tumor size and pathological type. Given the potential risks associated with AR, NAR could be considered as an acceptable and oncologically safe procedure for patients with early-stage ICC.

### Supplementary Information

**Additional file 1: Supplementary Table 1.** Study institutions and number of cases.

**Additional file 2: Supplementary Table 2.** Univariate and multivariate analysis of disease-free survival and overall survival in entire cohort.

**Additional file 3: Supplementary Table 3.** Previous studies comparing AR and NAR for patients with ICC.

**Additional file 4: Supplementary Fig 1.** Flow chart of patients’ selection.

## Data Availability

All data included in this study are available upon request by contact with the corresponding author.
