# Supplementary material for: Anatomic versus non-anatomic resection for early-stage intrahepatic cholangiocarcinoma: a propensity score matching and stabilized inverse probability of treatment weighting analysis
Source: BMC Cancer. 2023 Sep 11;23:850. doi: 10.1186/s12885-023-11341-z (PMC10496223; doi:10.1186/s12885-023-11341-z)
Supplement: Supplementary file 1 — Additional file 1: Supplementary Table 1. Study institutions and number of cases. [file 12885_2023_11341_MOESM1_ESM.docx]

| **Supplementary Table 1. Study institutions and number of cases** | |
| --- | --- |
| **Study institutes** | **Patients Number** |
| Mengchao Hepatobiliary Hospital of Fujian Medical University | 42 |
| Eastern Hepatobiliary Surgery Hospital | 127 |
| Affiliated Cancer Hospital of Chinese Academy of Medical Sciences | 15 |
| Tongji Hospital Affiliated to Tongji Medical College, Huazhong University of Science &Technology | 7 |
| Beijing Friendship Hospital Affiliated to Capital Medical University | 10 |
| Xuanwu Hospital Affiliated to Capital Medical University | 10 |
| Tiantan Hospital Affiliated to Capital Medical University | 5 |
| the Affiliated Hospital of Chuanbei Medical University | 3 |
| Renji Hospital Affiliated to Shanghai Jiaotong University | 12 |
| West China Hospital of Sichuan University | 16 |
| the Southwest Hospital Affiliated to the Army Medical University | 20 |
| the Second Affiliated Hospital, Zhejiang University School of Medicine | 11 |
