# Supplementary material for: Anatomic versus non-anatomic resection for early-stage intrahepatic cholangiocarcinoma: a propensity score matching and stabilized inverse probability of treatment weighting analysis
Source: BMC Cancer. 2023 Sep 11;23:850. doi: 10.1186/s12885-023-11341-z (PMC10496223; doi:10.1186/s12885-023-11341-z)
Supplement: Supplementary file 2 — Additional file 2: Supplementary Table 2. Univariate and multivariate analysis of disease-free survival and overall survival in entire cohort. [file 12885_2023_11341_MOESM2_ESM.docx]

| Supplementary Table 2. Univariate and multivariate analysis of disease-free survival and overall survival in entire cohort | | | | | | | | | | | |
| --- | --- | --- | --- | --- | --- | --- | --- | --- | --- | --- | --- |
| **Characteristics** | **Disease-free survival** | | | | |  | **Overall survival** | | | | |
|  | **Univariate** | |  | **Multivariate** | |  | **Univariate** | |  | **Multivariate** | |
|  | **HR (95CI)** | ***P-*value** |  | **HR (95CI)** | ***P-*value** |  | **HR (95CI)** | ***P-*value** |  | **HR (95CI)** | ***P-*value** |
| **Gender (**Male vs Female**)** | 0.68 (0.49-0.96) | 0.027 |  | 0.74 (0.53-1.05) | 0.089 |  | 0.75 (0.53-1.08) | 0.124 |  |  |  |
| **Age (**≥ 60 vs <60 years) | 0.96 (0.70-1.31) | 0.792 |  |  |  |  | 1.07 (0.76-1.50) | 0.687 |  |  |  |
| **CA19-9 (**>200 vs ≤200 U/mL**)** | 0.83 (0.51-1.34) | 0.443 |  |  |  |  | 1.13 (0.70-1.84) | 0.623 |  |  |  |
| **CEA (**>5 vs ≤5 ug/mL**)** | 0.88 (0.59-1.31) | 0.524 |  |  |  |  | 1.21 (0.81-1.82) | 0.353 |  |  |  |
| **ECOG score (**≥2 vs 0-1**)** | 0.81 (0.59-1.11) | 0.187 |  |  |  |  | 0.99 (0.70-1.41) | 0.975 |  |  |  |
| **Child-Pugh class (**B vs A**)** | 1.19 (0.87-1.62) | 0.278 |  |  |  |  | 1.17 (0.84-1.64) | 0.350 |  |  |  |
| **Intraoperative blood loss** (>400 vs ≤400 mL) | 0.77 (0.50-1.18) | 0.233 |  |  |  |  | 0.82 (0.51-1.32) | 0.417 |  |  |  |
| **Intraoperative transfusion (**Yes vs No**)** | 0.81 (0.53-1.27) | 0.361 |  |  |  |  | 0.92 (0.57-1.47) | 0.721 |  |  |  |
| **Operation time (**>180 vs ≤180 min**)** | 1.12 (0.86-1.45) | 0.631 |  |  |  |  | 1.15 (0.70-1.60) | 0.788 |  |  |  |
| **Major hepatectomy (**Yes vs No**)** | 1.13 (0.83-1.54) | 0.427 |  |  |  |  | 1.60 (1.13-2.26) | 0.008 |  | 3.19 (0.69-14.68) | 0.136 |
| **Surgical margin (**≥1cm vs <1 cm**)** | 0.51 (0.41-0.78) | 0.001 |  | 0.63 (0.46-0.88) | 0.007 |  | 0.66 (0.47-0.94) | 0.021 |  | 0.68 (0.47-0.99) | 0.042 |
| **Anatomic resection (**Yes vs No**)** | 0.85 (0.62-1.17) | 0.317 |  | 1.04 (0.75-1.44) | 0.811 |  | 1.09 (0.78-1.53) | 0.612 |  | 1.19 (0.83-1.70) | 0.354 |
| **Hospital stays (**>15 vs ≤15 days**)** | 1.09 (0.79-1.49) | 0.609 |  |  |  |  | 1.12 (0.80-1.58) | 0.512 |  |  |  |
| **Cirrhosis (**Yes vs No**)** | 1.02 (0.74-1.41) | 0.897 |  |  |  |  | 1.13 (0.80-1.59) | 0.489 |  |  |  |
| **Tumor diameter (**>5 vs ≤5 cm**)** | 1.06(0.78-1.44) | 0.721 |  |  |  |  | 1.54 (1.09-2.19) | 0.015 |  | 1.51 (0.11-2.37) | 0.389 |
| **Mass-forming (**Yes vs No**)** | 0.64 (0.47-0.87) | 0.005 |  | 0.71 (0.51-0.97) | 0.034 |  | 0.74 (0.52-1.04) | 0.085 |  | 0.79 (0.55-1.13) | 0.190 |
| **Tumor differentiation (**Well&Moderate vs Poor**)** | 0.76 (0.52-1.12) | 0.161 |  |  |  |  | 0.99 (0.66-1.47) | 0.943 |  |  |  |
| **Satellite (**Yes vs No**)** | 1.56 (0.88-2.75) | 0.127 |  |  |  |  | 1.77 (0.98-3.21) | 0.059 |  | 2.36 (1.24-4.51) | 0.009 |
| **Adjuvant Therapy (**Yes vs No**)** | 0.99 (0.66-1.47) | 0.942 |  |  |  |  | 0.83 (0.53-1.31) | 0.424 |  |  |  |
| **Note**: NAR, nonanatomic resection; AR, anatomic resection; HBV, hepatitis B virus; CA19-9, carbohydrate antigen 19-9; CEA, carcinoembryonic antigen; ECOG, the Eastern Cooperative Oncology Group. | | | | | | | | | | | |
