# Supplementary material for: Anatomic versus non-anatomic resection for early-stage intrahepatic cholangiocarcinoma: a propensity score matching and stabilized inverse probability of treatment weighting analysis
Source: BMC Cancer. 2023 Sep 11;23:850. doi: 10.1186/s12885-023-11341-z (PMC10496223; doi:10.1186/s12885-023-11341-z)
Supplement: Supplementary file 3 — Additional file 3: Supplementary Table 3. Previous studies comparing AR and NAR for patients with ICC. [file 12885_2023_11341_MOESM3_ESM.docx]

| **Supplementary Table 3. Previous studies comparing AR and NAR for patients with ICC** | | | | | | | | |
| --- | --- | --- | --- | --- | --- | --- | --- | --- |
| **Studies** | **Design** | **Population** | **Treatment** | **DFS rates (%)** | ***P*-value** | **OS rates (%)** | ***P*-value** | **Surgical factors and complications** |
|  |  |  |  | **1-, 3-, 5-year** |  | **1-, 3-, 5-year** |  |  |
| Li 2018  [20] | Retrospective single center | Solitary ICC without direct invasion to contiguous organs or extrahepatic metastasis | Before PSM:  AR: n= 85, NAR: n=65  After PSM:  AR: n= 29, NAR: n=29 | Before PSM  AR: 48.9/27.2/27.2  NAR: 50.9/32.8/32.8  After PSM  AR: 53.2/19.2/19.2  NAR: 58.6/41.0/41.0 | Before PSM:0.607  After PSM: 0.370 | Before PSM:  AR: 65.0/35.4/29.1  NAR: 75.2/47.0/25.7  After PSM:  AR: 70.2/22.9/22.9  NAR: 71.1/51.7/51.7 | Before PSM: 0.477  After PSM: 0.229 | The operation time, proportion of intraoperative transfusion, amount of blood loss, length of hospital stay and occurrence of complications were comparable between the AR and NAR groups. |
| Wu  2018  [32] | Retrospective single center | Primary ICC | AR: n=68  NAR: n=30 | - | - | Median OS:  AR: 14 months  NAR: 11 months | 0.031 | The AR group displayed a longer operation time, but the proportion of intraoperative transfusion, amount of blood loss, length of hospital stay and occurrence of complications were comparable between the AR and NAR groups. |
| Si 2019  [13] | Retrospective single center | AJCC I/II/III  ICC | Before PSM:  AR: n= 305, NAR: n=366  After PSM:  AR: n= 229, NAR: n=229 | Before PSM  AR: 57.7/35.7/29.1  NAR: 42.1/23.4/ 17.2  After PSM  AR:58.1/35.7/ 28.1  NAR:44.1/23.9/18.0 | Before PSM: <0.001  After PSM: 0.002 | Before PSM:  AR: 74.4/45.8/ 36.3  NAR: 61.2/30.5/24.2  After PSM:  AR: 72.9/45.7/36.0  NAR: 62.0/30.8/25.3 | Before PSM: <0.001  After PSM: 0.002 | The operation time, proportion of intraoperative transfusion, amount of blood loss, length of hospital stay and occurrence of complications were comparable between the AR and NAR groups. |
| Han 2023  [33] | Retrospective single center | Elder (65-79 years) patients with ICC | AR: n=87  NAR: n=94 | - | - | 1. year OS rate:   AR: 41.38  NAR: 25.53 | 0.008 | The AR group displayed a longer operation time, lower amount of blood loss and lower incidence of complications. |
| Wang 2022 [34] | Retrospective single center | Primary ICC | Before PSM:  AR: n= 139, NAR: n=137  After PSM:  AR: n= 99, NAR: n=99 | Before PSM:  AR: 58/19/7  NAR: 50/13/3  After PSM:  AR:61/21/10  NAR:49/12/4 | Before PSM: 0.131  After PSM: 0.029 | Before PSM:  AR: 68/44/28  NAR: 64/34/22  After PSM:  AR: 70/46/ 34  NAR: 60/28/16 | Before PSM: 0.195  After PSM: 0.023 | The AR group displayed a higher proportion of intraoperative transfusion and longer hospital stay, but the operation time, amount of blood loss, and occurrence of complications were comparable between the AR and NAR groups. |
| Wu 2023  [14] | Retrospective multi-center | Primary ICC lesions without  contiguous organ invasion or extrahepatic metastasis | Before PSM:  AR: n= 80, NAR:n=67  After PSM:  AR: n= 50, NAR: n=50 | Before PSM:  AR: 59.5/36.5/ 20.5  NAR: 38.2/12.1/ 6.9  After PSM:  AR:49.2/24.7/16.5  NAR:28.0/11.2/4.5 | Before PSM: <0.001  After PSM: 0.010 | Before PSM:  AR: 78.7/58.9/28.5  NAR: 61.2/25.4/8.8  After PSM:  AR: 65.8/50.1/22.5  NAR: 52.0/16.5/6.3 | Before PSM: <0.001  After PSM: 0.016 | The operation time, amount of blood loss, length of hospital stay and occurrence of complications were comparable between the AR and NAR groups. |
| Notes: ICC, intrahepatic cholangiocarcinoma; PSM, propensity score matching; AR, anatomic resection; NAR, nonanatomic resection. | | | | | | | | |
