# Supplementary figures and images for: Anatomic versus non-anatomic resection for early-stage intrahepatic cholangiocarcinoma: a propensity score matching and stabilized inverse probability of treatment weighting analysis
Source: BMC Cancer. 2023 Sep 11;23:850. doi: 10.1186/s12885-023-11341-z (PMC10496223; doi:10.1186/s12885-023-11341-z)

**Supplementary Fig 1.** Flow chart of patients’ selection.


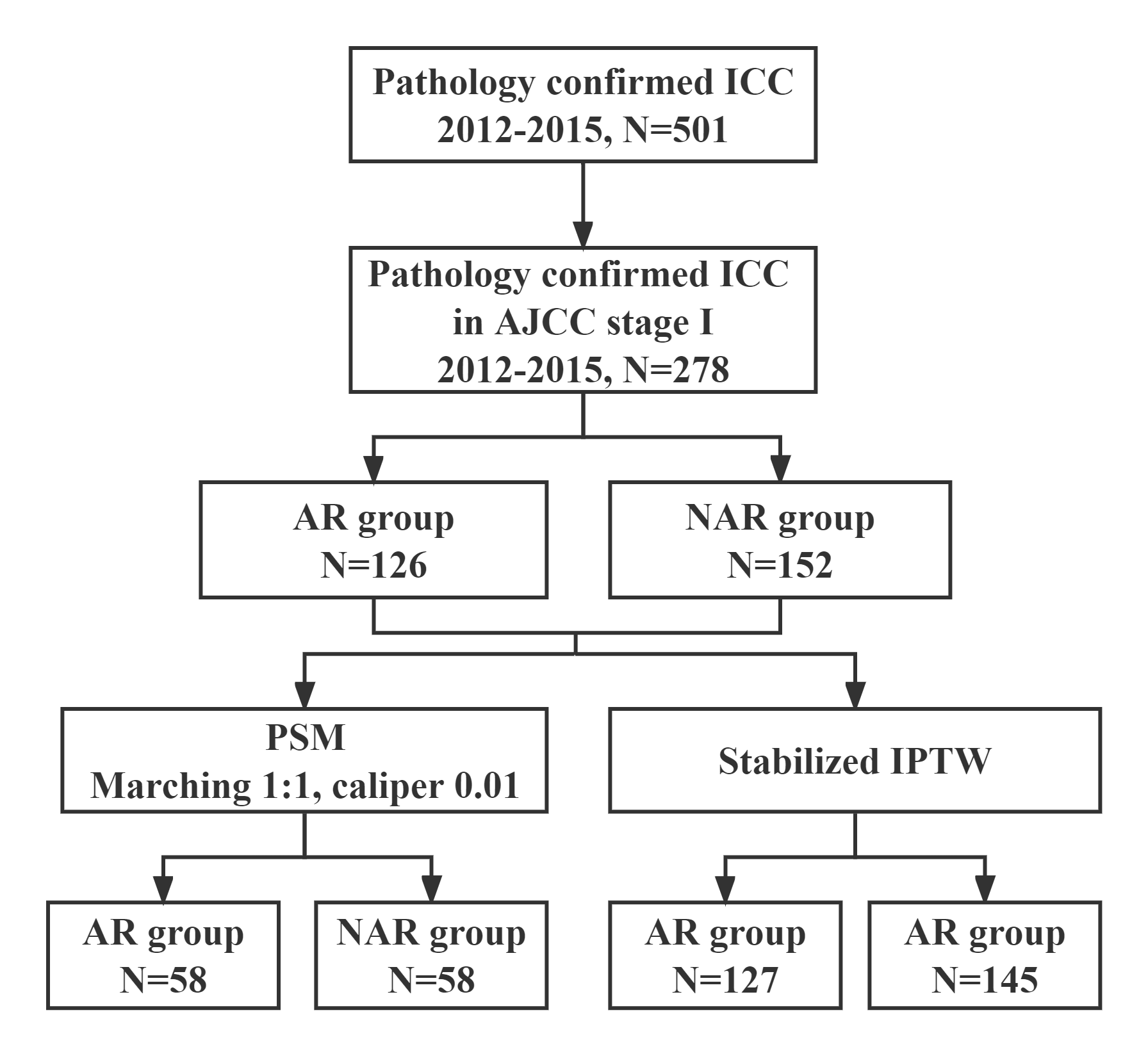

Supplement: Supplementary file 4 — Additional file 4: Supplementary Fig 1. Flow chart of patients’ selection. [file 12885_2023_11341_MOESM4_ESM.docx]
